# Supplementary material for: Understanding the Diurnal Oscillation of the Gut Microbiota Using Microbial Culture
Source: Life (Basel). 2023 Mar 19;13(3):831. doi: 10.3390/life13030831 (PMC10054680; doi:10.3390/life13030831)
Supplement: Supplementary file 1 [file life-13-00831-s001.zip › life-2188654-supplementary.pdf]

Table S1. Median (minimum-maximum) CFU/ml values, considering culture media, sample type, and collection time. Different capital letters in the same column represent statistically significant differences in alphabetic order among the collection sites for the same ZT ($p<0.05$; Kruskal-Wallis, Dunn). Different symbols in the same column represent statistically significant differences for different ZT specimens from the same site in the same culture media ($p<0.05$; Kruskal-Wallis, Dunn). BHI = Brain Heart Infusion Agar; SA = Sabouraud Agar; MS = Mitis Salivarius Agar; BA = Brucella Agar; FC = Feces; CE = Cecum; RT = Rectum; ZT = Zeitgeber time.

	**Feces Sample (FC)**			
	**BHI (CFU × 10^{+7})**	**SA (CFU × 10^{+2})**	**MS (CFU × 10^{+6})**	**BA (CFU × 10^{+7})**
ZT0	5.20 (0.15 – 17.20)	4.00 (0.80 - 8.00) **A**	156.00 (80.00 - 172.00) **A**	30.00 (30.00 – 30.00) *****
ZT6	20.00 (0.30 – 20.80) **A**	28.00 (0 - 52.00)	180.00 (3.00 - 300.00)	18.00 (0.10 – 30.00) #
ZT12	9.20 (7.20 – 14.80) **A**	0 (0 - 4.00)	80.00 (64.00 - 12.00)	0.05 (0.001 – 0.18) **Ø**
ZT18	10.00 (6.00 – 14.00)	124.00 (0 - 400.00)	84.00 (52.00 - 300.00)	0.30 (0.21 – 5.20) **Ɵ**
	**Cecum Sample (CE)**			
	**BHI (CFU × 10^{+7})**	**SA (CFU × 10^{+2})**	**MS (CFU × 10^{+6})**	**BA (CFU × 10^{+7})**
ZT0	0.23 (0.04 – 4.40)	0.40 (0.40 - 0.80) **B**	32.00 (0.76 - 76.00) **B**	30.00 (0.12 – 30.00)
ZT6	6.00 (0.30 – 12.80) **B**	12.40 (4.800 - 44.00)	20.00 (2.40 - 64.00)	0.30 (0.16 – 7.20)
ZT12	0.21 (0.11 – 4.80) **B**	5.60 (0 - 8.00)	32.00 (28.00 - 300.00)	0.003 (0.001 – 22.80)
ZT18	0.27 (0.08 – 6.80)	3.60 (0 - 5.20)	3.00 (2.04 - 300.00)	0.14 (0.00004 – 30.00)
	**Rectum Sample (RT)**			
	**BHI (CFU × 10^{+4})**	**SA (CFU × 10^{+2})**	**MS (CFU × 10^{+5})**	**BA (CFU × 10^{+7})**
ZT0	1.80 (0.002 – 22.00)	0 (0 - 0.40) **C**	0.17 (0.0016 - 14.00) **C**	12.00 (0.03 – 30.00)
ZT6	116.00 (0.30 – 17.20) **C**	0.80 (0.40 - 10.80)	3000.00 (2240.00 - 3000.00)	11.60 (0.30 – 19.60)
ZT12	16.00 (0.30 – 4.80) **C**	0 (0 - 0)	4.40 (2.80 - 3000.00)	8.40 (0.23 – 13.60)
ZT18	80.00 (2.80 – 20.80)	0 (0 - 48.00)	3000.00 (30.00 - 3000.00)	14.40 (12.00 – 14.40)
